# Dr. Miller’s Latest Investigation

**Published:** 1883-12

**Authors:** 


### DR. MILLER'S LATEST INVESTIGATION.

The leading article in this number contains the results of the later observations and studies of Dr. Miller, in Etiology. We do not need to call attention to it, for members of the dental profession everywhere, read with interest whatever he writes. His views are not merely theoretical and speculative, but are the conclusions deduced from such a series of experiments as very few men have entered upon. He expresses himself clearly and lucidly, and gives all the data upon which his inferences are founded.

Whether he concurs with him or holds different views, no candid man can withhold the tribute of respect and admiration for such a tireless student and patient observer. The whole dental world is under obligations to him for the perseverance with which he has labored to unravel the mystery in which is shrouded so much of oral pathology.

Nor has he yet found a stopping-place. A letter but recently received, speaks in hopeful strains of the results of his very latest series of experiments, not yet concluded. He says: "I have discovered and successfully cultivated a fungus *always* present in the human mouth and in carious dentine, whose development is, under proper conditions (such as are constantly found in the mouth), always accompanied by the production of a strong acid. I have also determined the conditions favorable and unfavorable to its development, and have nearly finished the analysis of its acid. To ascertain this I have made over *three hundred* experiments and cultures. I am very hopeful that this will furnish a key to the solution of the whole problem."

Dr. Miller's article containing an account of these investigations and the conclusions to which they have led him, will appear in the INDEPENDENT PRACTITIONER early in the coming year.

